# Quality of Root Canals Performed by the Inaugural Class of Dental Students at Libyan International Medical University

**DOI:** 10.1155/2015/135120

**Published:** 2015-06-08

**Authors:** Ranya F. Elemam, Ziad Salim Abdul Majid, Matt Groesbeck, Álvaro F. Azevedo

**Affiliations:** ^1^Department of Restorative Dentistry and Periodontology, School of Dentistry, Libyan International Medical University (LIMU), Benghazi, Libya; ^2^University of Porto, Porto, Portugal; ^3^Salt Lake City, UT, USA; ^4^Faculty of Dentistry, EPIUNIT-ISPUP,, University of Porto, Porto, Portugal

## Abstract

*Objective*. The purpose of this study was to radiographically evaluate technical quality of root canal fillings performed by dental undergraduates at Libyan International Medical University in Libya. *Methods*. Root canal cases were treated at university dental clinic from the fall of 2012 to the fall of 2013 by the fourth and fifth year dental students. Students used step-back preparation and cold lateral compaction in the treatment. Radiographs were reviewed over a two-year period from initial procedure to final restoration. Radiographs were evaluated for adequacy or inadequacy by length, density, and taper. Length inadequacy was classified as short or overextended. Overall quality was considered “adequate” based on all three variables. Chi-square tested differences between teeth groupings and adequacy classification. Significant *p* value results were adjusted by Bonferroni correction. *Results*. Adequate length of root canal fillings were observed in roughly half of all samples (48.6%). Density was adequate in 75.8% of the samples. Taper was observed as adequate in 68.8%. Higher quality was evident in anterior teeth (plus premolars) versus molars (65.6% versus 43.3%, resp.; *p* < 0.04). *Conclusion*. Overall quality of endodontic treatment performed by undergraduate dental students was adequate in 53.9% of the cases. Significant opportunity exists to improve the quality of root canals provided by dental students.

## 1. Introduction

Teaching undergraduate endodontics has been recognized as one of the most formidable challenges across all dental subjects [1]. Educators have had to continuously cope with the discipline's contemporary evolution, which has rapidly spread in the past 2 decades and even outpaced other dental specialties by measures of scholarly research activity [2]. The foremost educational goal of endodontics is to successfully promulgate knowledge as a foundation for graduates to become competent and proficient in actual practice [3]. All endodontic treatment modalities require advanced knowledge and technical skills should be considered essential in pursuing this objective [4].

In contemporary endodontic curricula, educators have devoted special focus to optimize technical quality of root canal procedures. Some studies have demonstrated an association between root canal-specific training during the student's study period and improved quality of root canal fillings performed by dental graduates [5–8]. Further efforts have been made to improve root canal quality via postgraduate interventions, including continuing dental education (CDE) or development of a quality improvement initiative to improve quality of care [6, 9].

The European Society of Endodontology (ESE) has published undergraduate curriculum guidelines updated every decade to encourage the development of high quality undergraduate dental education and acceptable standards of care in clinical endodontic practice [3, 10–12]. These guidelines, widely integrated into endodontic curricula [1], emphasize the necessity for undergraduate students to undertake principles of clinical and theoretical education and apply them to the clinical outcome to reach a minimum competency threshold prior to graduation. Because root canals are widely performed by general dental practitioners as opposed to specialists alone, guiding principles place high expectation for dental students to demonstrate a satisfactory nonsurgical root canal procedure on both single- and multirooted teeth.

We have directly observed endodontic practical sessions in Libya's government-run dental schools and found that they offer abbreviated and very limited exposure to endodontic topics that are inadequate to cultivate knowledge and competence. Reasons for this suboptimal training likely include (1) the vast number of dental students, (2) fewer available patients, (3) a sparsity of endodontic equipment and material availability, (4) limited endodontics staff, and (5) the prevailing belief that endodontics should be a specialist subject. The absence of complete endodontic training in dental education may therefore severely impair a graduate's decision-making and clinical effectiveness, leading to pervasive treatment failures.

In the fall of 2007, the first accredited private medical university in Libya was founded in Benghazi, Libyan International Medical University (LIMU), with the mission to graduate highly qualified graduates in different areas of health. LIMU is the only educational alternative to government-run schools for prospective students in Benghazi. The LIMU dental curricula mandate that endodontic training should be provided to all dental students within five years. A preclinical course is tentatively started in the third year requiring students to perform root canals on at least four anterior human-extracted teeth, two premolars, and two molars. Clinical courses follow, with fourth and fifth year dental students undertaking education in endodontic treatment tailored to specific requirements, including a comprehensive clinical examination informing appropriate diagnosis. During the fourth year, students perform the nonsurgical root canal treatment of four anterior teeth and four premolars. In the fifth year, students are required to complete primary endodontic procedure of three molars in the first semester and a comprehensive case treatment in the second semester. In the sixth year's nine month internship period, students undertake routine orthograde root canal therapy per patient presentation.

Research evaluations of root canal treatment quality have been shown to significantly aid the planning of future endodontic educational programs [13]. While quality evaluations of root canals performed by graduated students during their preclinical and clinical coursework have been widely reported elsewhere, there have been no published reports originating from Libya regarding quality of root canal fillings performed by dental students.

The aim of this study was to evaluate the technical quality of root canal treatment performed by the first undergraduate group during their clinical academic terms in both the fourth and fifth years at LIMU. This effort was also undertaken to gauge the scope of revisions necessary to successfully modify the preclinical program curriculum delivered during the preclinical semesters.

## 2. Methods

### 2.1. Patients

Patient cases were treated by thirty-two undergraduate students in the university dental clinic during their fourth and fifth years from the fall of 2012 to the fall of 2013. The study protocol was reviewed and exempted by the institutional review board. All students were supervised by staff specialized in endodontics in the first clinical year. In the final year, a conservative specialist with interest in endodontics was appointed. The ratio of clinical supervisor to student was 1 to 8.

All chart records and radiographs of patients who had received endodontic student treatment at LIMU were collected and reviewed by an investigator from initial procedure time to final restoration, all over the two-year academic period following the group of thirty-two dental students through their clinical course. A total of twenty-seven dental students entered their fifth year, reflecting minor expected attrition. Patients were excluded if they were younger than 16 years of age and if they had records that did not include preoperative and postoperative periapical radiographs, unreadable radiographs due to developing procedures, superimposed anatomical structures, records without complete root canal treatment, or cases of perforation, instrument separation, or missing canals. In year four, the thirty-two students treated a total of 256 teeth. In year five, the twenty-seven remaining students treated 81 teeth. Thus, a total of 337 teeth were treated over the two-year academic period.

### 2.2. Procedure

After assessing the medical and dental history of each patient, local anesthesia was administered using 2% lignocaine 1 : 20,000 (Alexandria Co., Alexandria, Egypt). Rubber dam isolation was used for all patients. Access cavity was prepared and the working length was determined using size of 15 K file (Dentsply, Dentsply Ltd., UK). Periapical radiographs were then taken using the paralleling technique with Trophy (France) X-ray unit and the Kodak D-Speed films were exposed at 65 kV, 10 mA. Step-back technique using a stainless steel hand K-files (0.02 taper) was performed and root canals were irrigated using 1% sodium hypochlorite (NaOCl). EDTA 17% gel was used to negotiate calcified canals. All root canals were filled with gutta-percha 0.02 taper (Gapadent co., Ltd., Hamburg, Germany) and zinc oxide-based sealer (Metabiomed Co., Ltd., Korea) using cold lateral condensation technique. An NiTi finger spreader of 2% taper was used to compact the gutta-percha cones and create a space for accessory points. For each root-filled tooth, at least 2 periapical radiographs were taken (preoperative and postobturation). One investigator, a specialist in endodontics, independently examined the radiographs utilizing a magnifying lens (×4) and an X-ray viewer.

### 2.3. Outcome Variables

Technical quality of root canal fillings was assessed by radiography based on 3 variables: (1) length as compared to the radiographic apex, (2) density of obturation by the presence or absence of voids, and (3) taper. The density and taper of root canal fillings were classified as adequate or inadequate. Length was rated as adequate or short (inadequate) or overextended (also inadequate). Overall quality was deemed “adequate” if all 3 variables were acceptable according to protocol-specified criteria as presented in Table 2. Postobturation radiographs were captured via paralleling technique, displaying the entire length of the root and 2 to 3 mm beyond it (see sample radiographs in Figures 5(a), 5(b), and 5(c)). Radiographs were assessed for adequate length quality (see the example in Figure 5(a)). Figures 5(b) and 5(c) represented inadequate quality that was either too short in length or of overextended length, respectively.

### 2.4. Statistical Analysis

Descriptive statistics present categorical variable frequencies and percentages. Chi-square test statistic was performed to determine statistically significant differences between the “adequate” and “inadequate” counted variables. Alpha level was set at .05. If significance was reached, Bonferroni correction was applied to adjust the *p* value by multiplying it by the number of the comparisons (2 + 1) in each maxillary and mandibular teeth to account for multiple comparisons. Both Excel 2013 (Microsoft, Redmond, WA, USA) and SPSS software v.20 (SPSS Inc., Chicago, IL, USA) were used for all statistical procedures and validation of analysis.

## 3. Results

After applying inclusion and exclusion criteria, 128 teeth constituted the final sample over the two-year academic period (32 anterior teeth, 29 premolars, and 67 molars).

### 3.1. Overall Quality (Figure 1)

#### 3.1.1. Maxilla and Mandible Combined

**Figure 1:**
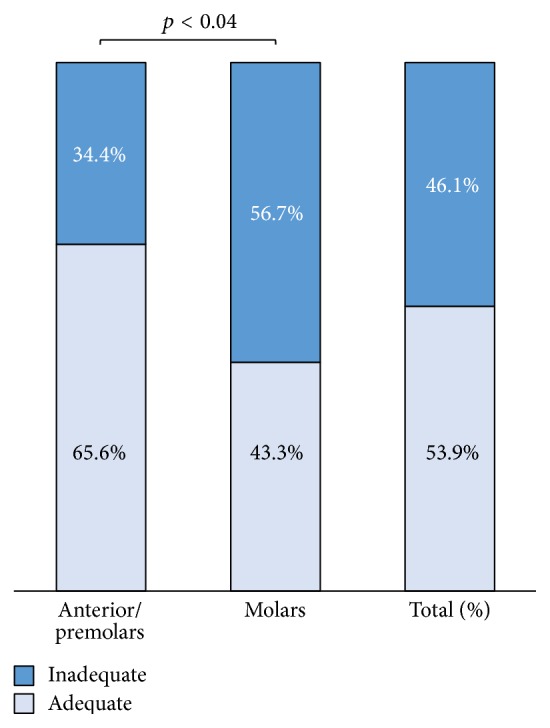
Overall quality (%) of root canals in maxillary and mandibular teeth.

**Figure 5:**
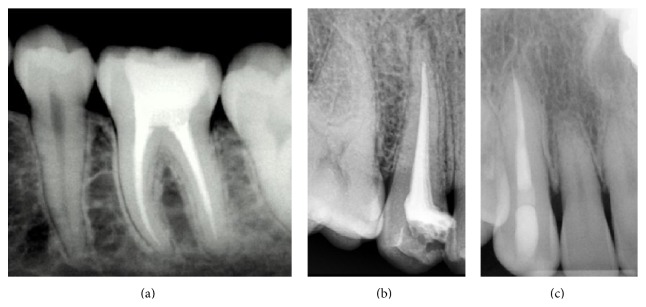
Radiographs depicting adequate quality root canal (a), inadequate/short length (b), and inadequate/overextended length (c).

The overall quality was defined by the combination of all three outcome variables that were deemed adequate (length, density, and taper) for all maxillary and mandibular teeth. All measures of adequacy are reported in this results' section and in Figures 1 through 4, whereas inadequate percentages are only reported in the same corresponding figures.

Overall quality was deemed adequate in 53.9% of all maxillary and mandibular teeth. There were statistically significant differences between teeth types, with 65.6% of anterior teeth and premolars classified as adequate compared to 43.3% of molars (*p* < 0.04; Figure 1). By outcome variable for both maxillary and mandibular teeth combined, root canal filling length was observed to be adequate in 48.6% of all teeth, density was adequate in 75.8%, and taper was adequate in 68.8%.

### 3.2. Length (Figure 2)

#### 3.2.1. Maxilla

Adequate length of root filling was observed in 64.0% of all maxillary teeth. The incisors and molars demonstrated fewer numbers of teeth with adequate length compared to canines and premolars, although this difference was not statistically significant (*p* > 0.2).

#### 3.2.2. Mandible

For mandibular teeth, only premolars and molars were existing and thus analyzed. Adequate length was observed in 33.3% of all mandibular teeth, of which 37.5% were premolars and 32.4% were molars, but the difference between these groups was not found to be statistically significant (*p* > 0.7).

### 3.3. Density (Figure 3)

#### 3.3.1. Maxilla

Adequate density of root filling was seen in 82.6% of all maxillary teeth, 57.6% of which were molars and 98.1% were anterior teeth plus premolars. When both groups were compared, the difference was found to be statistically significant (*p* < 0.0001).

#### 3.3.2. Mandible

Adequate density was observed in 69.0% of all mandibular teeth. All premolars were adequate (100%), in contrast to 61.8% of the molars, and the difference was also statistically significant (*p* < 0.1).

### 3.4. Taper (Figure 4)

#### 3.4.1. Maxilla

Adequate taper was found in 68.6% of all maxillary teeth. The maxillary canines that demonstrated the highest rate of taper (87.5%) compared to the molars were (54.5%), but this difference was not found to be statistically significant (*p* > 0.1).

#### 3.4.2. Mandible

Adequate taper was found in 69.0% of all mandibular teeth, 87.5% of which were premolars and 64.7% were molars, but this difference was also not statistically significant (*p* > 0.05).

## 4. Discussion

This is the first endodontic research study of its kind reported from Libya. These data objectively identify the quality of endodontic treatments performed by Libyan dental students, who would shortly be expected to serve the community. Other studies radiographically assessed only the length and density of root canal filling but omitted the taper variable [14–17]. We incorporated the taper variable defined by guidelines [18] and results were comparable to other research studying tapers [5, 14, 15, 19]. We observed adequate taper in 68.8% of root canals, a finding slightly less than the 71% reported by Román-Richon and colleagues, in which rotary files were used and 82% were reported by Fonseka and colleagues, of which the latter study only investigated single-rooted molars with wide versus narrow canals [15, 19].

Our percentage of overall quality was 53.9%, less than the reports from Turkey (79.5%), Serbia (74%), and Malaysia (61%) [16, 17, 20], similar to Greece (55%) [21], but greater than reports from Iran (45%), Spain (44%), Sudan (24%), and Saudi Arabia (23%) [5, 13, 14, 19]. However, inequalities may be difficult to reconcile because of the differences in outcome criteria used, sample sizes, and design. We report a low percentage of adequate molars treated in the second year (43%) compared to the adequately-treated anterior and premolars in the first year (65.6%), which skewed the overall percentage of adequate root fillings to 53.9%. Difficulty in treating molars by undergraduate students was also reported in other studies [13, 14, 20, 21]. In contrast, a Sudanese study showed that the adequacy of posterior teeth was 79.7% versus 20.3% of anterior teeth [5]. One plausible explanation to account for this discrepancy might be attributable to experiential learning and progressive adaptation of individual students.

Overall, inadequate root canal fillings were seen in 46.1% of root canals (Figure 1). Molars showed high percentage of inadequacy 56.7%, which was significantly different from 34.4% of anterior teeth (plus premolars). Difficulty in successfully treating molars was managed by most dental schools [21, 22]. In our study, this significant value may be attributable to the anatomical complexity of molars, lack of specialist supervision during treatment of molars, and insufficient training in time and depth of material devoted to molars and complication management in the preclinical curriculum.

Acceptable filling length was observed in 48.6% of all teeth. This was lower than most of the previous studies [13–17, 19]. This low value may be relevant to a high percentage of fillings with inadequate length in molars. To improve the length of root canal fillings, an electronic apex locator should be used in conjunction with X-ray radiographs. A homogeneous filling was found in 75.8% of root canals. This was less than that reported in other studies from Turkey (92%) [16] and Serbia (92.6%) [17]. In contrast, it was greater than that reported from Saudi Arabia and Iran (34%), Sudan (45%), and Spain (69%) [5, 13, 14, 19]. This might be explained by the fact that LIMU students were using Nitti finger spreaders, which have proven to provide better outcomes for lateral compaction technique, especially in curved canals because of deeper penetration [23].

It is important to recognize that improvements for greater educational impact can likely be bolstered by improved teacher-student alignment, credibility, trust, and a willingness to interact. Knowledge and competency are progressive achievements on the spectrum of learning toward mastery (expertise) [24]. Guidelines have suggested that students be supervised by appropriate endodontic specialists [12]. Our program employs an endodontic specialist in the first clinical year as a supervisor and a restorative specialist in the second. Past guidelines have also recommended an acceptable ratio between supervisor and students [25], which can aid in identifying student weaknesses [26]. Our supervisor-to-student ratio was 1 to 8 for both clinical years; ratios of other studies were 1 to 5 in Iran [13], 1 to 6 in Sudan [5], 1 to 12 in Spain, and 1 to 15 in Greece [27].

Our ratio allowed close monitoring and evaluation, both elements are instrumental in detecting student strengths and weaknesses. However, merely identifying student weaknesses and mishaps without prompt correction may inadvertently reinforce erroneous practices [28]. Even if supervisor feedback is immediate, manner of supervisor feedback is equally important, since competency can only be cultivated in conditions of constructive, directive feedback [12].

In our program and others, there are opportunities to optimize supervisor interaction with students, particularly by training supervisors in tactful approaches to a range of personality types that can ensure that students are motivated to actively participate [29]. Ideally, the supervisor should facilitate reflection of what the student has already learned, encouraged to self-evaluate their own weaknesses and, in opportune moments, remind students of acceptable practice standards [30]. A 2013 meta-analysis reported that planned, structured debriefings of either individuals alone or in teams can yield up to a 25% increase in performance regardless of it being a real case or a simulated setting [31]. Overall, effective learning is more likely to occur when a student is motivated to acknowledge that they are in need of input ultimately accepting direction [30, 32].

Instead of learning passively by simply being corrected, it is desirable for a student to be as active a learner can be as possible [33, 34]. A 2013 survey of dental students' perspectives reported that 92% preferred dynamic, interactive educational techniques [35]. Active learning can be even achieved by modifying traditionally didactic courses in breakout format for more group- or case-based discussions [36, 37], theater format to better visualize procedures [38], or interacting via an audience response system [18, 39]. It is incumbent upon educators to engage students while it is the responsibility of the students to be willing to participate.

Discerning and addressing student strengths and weaknesses are a key function of formative and summative evaluations, thereby informing educators of their student's needs to effectively model or redesign curricula. These processes are highlighted by both recent guidelines [12] and several studies reporting their use in medical education setting [29, 37, 40, 41] and academic teaching staff should accept the responsibility to employ formative and summative assessment techniques to determine priorities and then revise curricula accordingly just as other programs have to meet the needs of their students [21]. Direct observation of trainees is a vital hallmark of assessment to inform curricula across all specialties of medicine, including endodontic training [29]. As such, the supervisory role of educators, openness to student feedback, and the reporting of that feedback is crucial for actionable evaluations. This is even more important as emerging technologies might at any moment be adopted that could impact diagnosis, treatment, and the dissemination of real clinical skills in clinics or simulators [37, 40]. Qualtrough has estimated that students may be using haptic technology in practical learning applications as early 2020 [1]. Data from our study was compared to other recent published studies and revealed that international agreement related to student performance and the basic principles in applying European endodontics guidelines were followed. However, to improve success with molar teeth, preclinical training must be improved to acquire the clinical skills needed to treat molars. While problems in treating molars were noticed instantly by the endodontic staff at LIMU, a modified curriculum has already been implemented for subsequent groups.

## 5. New Curriculum Model

Our curriculum has undergone a number of changes and will likely be further revised. The practical sessions of the preclinical course has been divided between two semesters: the first semester of the third year (fifth semester) and the second semester of the fourth year (sixth semester). In the fifth semester, students treat at least four anterior teeth, two premolars and two molars, while in the sixth semester, they treat four molars. The competency-based method [42] was emphasized by ESE around the importance of student quality performance versus quantity [12]. However, there exists diversity between schools regarding minimum requirements of treated cases. The ESE sets minimum number of clinical experiences to be greater than twenty canals, including extracted teeth [43] (Table 1). In the LIMU curriculum, the total number of canals in the preclinical stage was set at 20 to 24.

In this added course, students received a one hour endodontic lecture and gained four hours lab experience per week over sixteen weeks dedicated almost exclusively for molars. During lab sessions, students provided a checklist for self-evaluation for each tooth. They were asked to finish root canal treatment in four extracted molars (two maxillary and two mandibular molars). To enhance their understanding toward the procedural errors and how to avoid them, students were asked to identify and document mishaps as soon as they occurred and to correct simple ledges and perforations. Students also performed treatment in single-rooted teeth using a rotary system for canal preparation. Later, they were asked to present one of their cases to explain the procedure and to detail mishaps and protective actions taken. Because a change took place in the preclinical course, future studies evaluating the clinical performance for this group of students are needed to investigate the impact of this newly-revised curriculum.

## 6. Conclusion

The quality of root canal fillings performed by undergraduate dental students at the Libyan International Medical University was satisfied in 53.9% of the cases, revealing a substantial gap in unmet educational needs. We must continue to adapt our educational plans to bolster student knowledge and confidence, particularly in treating molars, with the aim of ultimately yielding demonstrated improvements in competency. Testing the effect of the new model and several other education improvement initiatives are required to improve actual clinical performance of subsequent groups of dental students.

## Figures and Tables

**Figure 2:**
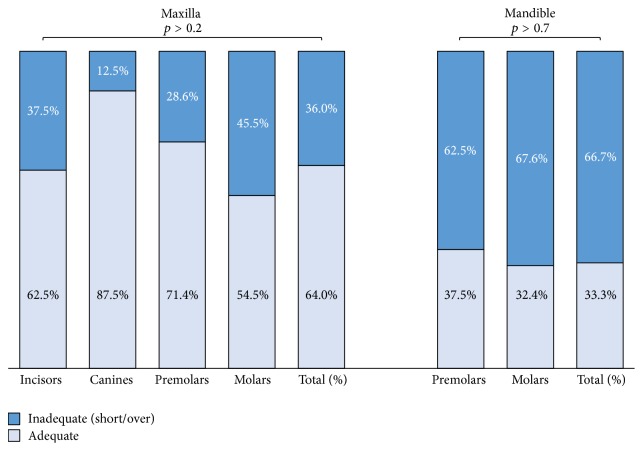
Length quality (%) in maxillary and mandibular root canal-filled teeth.

**Figure 3:**
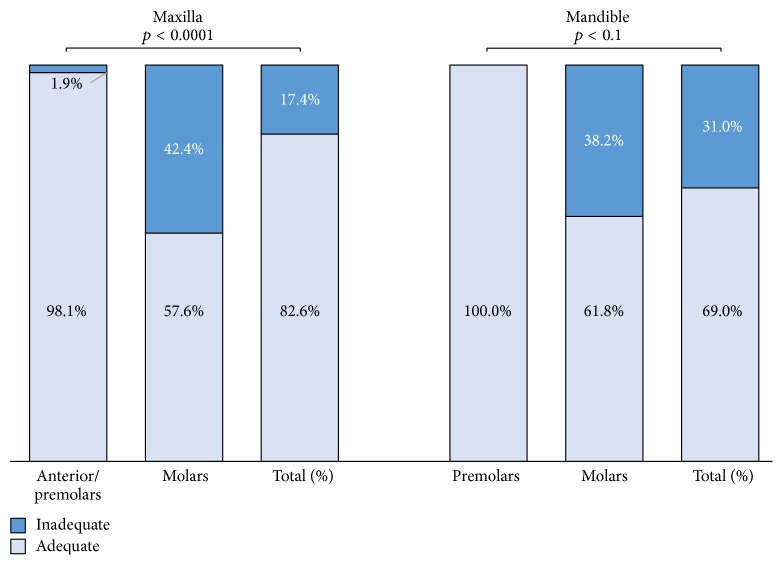
Density quality (%) in maxillary and mandibular root canal-filled teeth.

**Figure 4:**
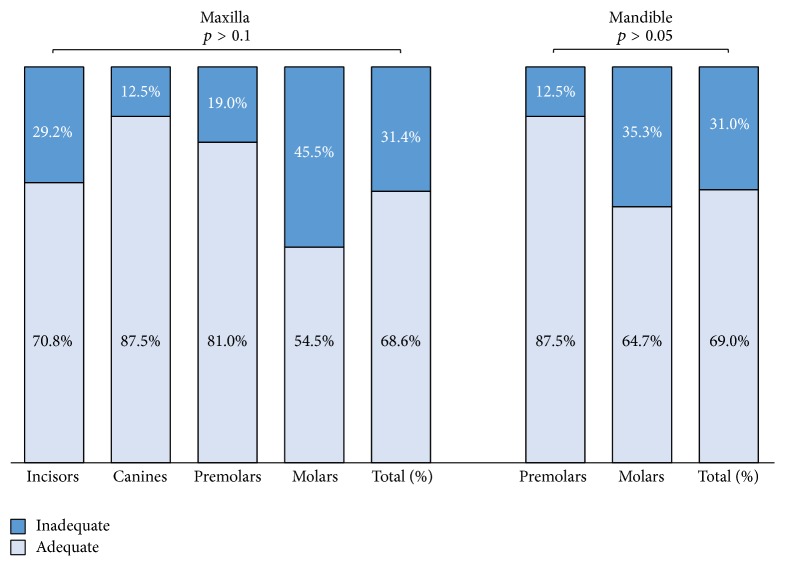
Taper quality (%) in maxillary and mandibular root canal-filled teeth.

**Table 1:** Distribution of teeth and root canals in both jaws.

	Incisors		Canines		Premolars		Molars	
	Teeth	Canals	Teeth	Canals	Teeth	Canals	Teeth	Canals
Maxillary	24	24	8	8	21	36	33	102
Mandibular	0	0	0	0	8	8	34	106

**Table 2:** Criteria for the evaluation of root canal fillings.

Quality variable	Criteria	Definition
Length	Adequate	Filling ends are 0.5 to 2 mm from the radiographic apex
	Overfilled	Filling extends beyond the radiographic apex
	Underfilled	Filling ends are shorter than 2 mm from the radiographic apex

Taper	Adequate	A consistent taper from coronal to apical aspects with good canal shape
	Inadequate	Inconsistent taper

Density	Adequate	Uniform density without clear presence of voids
